# Emergency Endoscopic Endonasal Optic Nerve Decompression for Graves' Orbitopathy

**DOI:** 10.7759/cureus.68384

**Published:** 2024-09-01

**Authors:** Omaima Qassab, Naouar Ouattassi, Dounia Kamal, Najib Benmansour, Mohamed Nourreddine El Alami El Amine

**Affiliations:** 1 Otolaryngology - Head and Neck Surgery, Sidi Mohamed Ben Abdellah University, Fez, MAR; 2 Otolaryngology - Head and Neck Surgery, Hassan II University Hospital of Fez, Fez, MAR; 3 Oral and Maxillofacial Surgery, Sidi Mohamed Ben Abdellah University, Fez, MAR; 4 Oral and Maxillofacial Surgery, Hassan II University Hospital of Fez, Fez, MAR; 5 Otolaryngology - Head and Neck Surgery, Sidi Mohamed Ben Abdellah University-, Fez, MAR

**Keywords:** minimally invasive, endonasal approach, endoscopic decompressive surgery, optic nerve, proptosis, graves’ orbitopathy

## Abstract

Graves' orbitopathy (GO) is a rare autoimmune disease that affects patients in their fourth to sixth decade, resulting in retro-orbital inflammation and hypertrophy of extraocular muscles and orbital fat. It is the most common disease affecting the orbit globally, and treatment options vary depending on the severity and activity status of the affection, ranging from local measures such as lubricating eye drops and patching, glucocorticoid eye drops, mydriatics, nonsteroid anti-inflammatory medications to systemic glucocorticoids, and emergency orbital decompression surgery. Immunotherapy and orbital radiation may as well be used as a treatment option even though their efficiency remains controversial. This paper presents the cases of two patients with GO who underwent endoscopic endonasal decompressive surgery. These patients' medical records, including symptoms and duration, clinical examination, imaging results, preoperative preparation, surgery steps, and postoperative course and outcomes, were collected from various specialties, including ophthalmologists and endocrinologists. We highlight the importance of a multidisciplinary approach to managing GO and its complications, with endoscopic endonasal techniques emerging as a minimally invasive and effective way to treat compressive optic nerve forms of the disease. However, the timing of decompression remains crucial, and early intervention is recommended to avoid sight-threatening ophthalmopathy when medical therapies are ineffective.

## Introduction

Graves' orbitopathy (GO), also known as thyroid-associated ophthalmopathy or thyroid eye disease (TED), is a vision-threatening orbital pathology that results from an autoimmune inflammatory disorder associated with thyroid disease, affecting visual and orbital tissues [1]. It primarily occurs in patients with overactive thyroid gland activity due to Graves' disease [1], but it can also manifest in cases of normal gland function or some cases of chronic autoimmune thyroiditis [2].

GO is generally considered an uncommon disease [3,4], with an estimated incidence of 16 women or three men per 100,000 people per year [5]. Severe forms are even less common, accounting for only 3% to 5% of all thyroid eye disease cases [6].

Clinically, various degrees of eye proptosis, oculomotor disorders, and eyelid abnormalities characterize GO. Severe forms may result in blindness due to compressive optic neuropathy or dangerous corneal ulceration. While GO is typically bilateral, it can clinically present unilaterally; however, radiological imaging usually confirms asymmetric bilateral disease [7]. GO typically progresses through two stages: an initial dynamic and progressive phase followed by a stable inactive phase.

Bilateral ocular symptoms and hyperthyroidism commonly occur simultaneously or within 18 months of each other. Sometimes, GO may precede or follow the onset of hyperthyroidism by several years [8]. While the management of thyroid pathology is a routine procedure, monitoring and controlling TED are crucial. We report the demographic, clinical, and management procedures of two patients who underwent surgery in our department for GO and discuss the clinical and therapeutic challenges involved.

The advantages of endoscopic endonasal decompression are multifaceted, making it a compelling approach in the realm of medical interventions. Firstly, the minimally invasive nature of the procedure is associated with a reduced morbidity, emphasizing patient safety and a quicker recovery. The precision afforded by endoscopic visualization allows for targeted decompression, specifically addressing proptosis and optic nerve compression in cases of GO. Additionally, the procedure boasts a distinct advantage in terms of aesthetics, as it eliminates the need for exterior incisions, thereby avoiding visible scars. This not only addresses cosmetic concerns but also contributes to a more positive postoperative experience for patients. Overall, endoscopic endonasal decompression represents a technologically advanced and patient-friendly solution, aligning with contemporary medical preferences for procedures that prioritize efficacy, minimal invasiveness, and aesthetic outcomes.

## Case presentation

Data collection

We collected data from the files of the two patients who underwent surgery for severe GO using an endoscopic approach. The data collected included patients’ symptoms, medical history, clinical otolaryngology, ophthalmology and endocrinologist examination results, preoperative imaging, and postoperative outcomes.

Preoperative work-up and preparation

The preoperative assessment included routine ORL examination with cervical and thyroid gland examination, upper aerodigestive tract examination, maxillo-facial examination to evaluate the degree of proptosis, ocular motility, and full cranial nerve exam. An ophthalmologist performed an eye examination, which included visual acuity, pupil reactivity, extraocular motility and alignment, intraocular pressure (IOP), confrontation visual fields, external examination (eyelids, lagophthalmos, and measure proptosis), routine slit-lamp examination, and fundoscopy. An orbital and paranasal sinus CT scan was performed to arrange the most suitable surgical approach and to precisely assess the anatomy of the spheno-ethmoidal complex (the Onodi cells, ethmoid septum -the perpendicular plate of the ethmoid representing a part of the posterior portion of the nasal septum-, etc.).

Topical treatments such as lubricating and glucocorticoid eye drops were prescribed to all the patients. Systemic therapies including intravenous (IV) glucocorticoids were used as a first-line treatment since our patients were considered as severe cases with active GO (the clinical activity score (CAS*) was more than 5/7 in both cases). On the other hand, oral glucocorticoids were prescribed for our patients mainly after starting a therapy phase with a number of systemic infusions with a dosage of 1 mg/kg body weight for a short period. Rituximab and/or tocilizumab were not used for our patients as well as orbital radiation.

As part of the preoperative preparation, patients were seen by an endocrinologist, and thyroid function tests were ordered. The results of TSH, T4, and T3 tests were within the normal range.

The CAS* is calculated according to the presence or absence of the following components: spontaneous retrobulbar pain; pain with eye movement; redness of the eyelids, redness of the conjunctiva, swelling of the eyelids, swelling of the caruncle and conjunctival edema or chemosis. The scores range from 0 to 7, with 0 to 2 indicating inactive GO and 3 to 7 indicating an active GO [2].

Postoperative care

The patient was prescribed a short-term course of antibiotics along with pain medication available on demand. Regular nasal saline irrigation was recommended, preferably using a warm saline solution, and de-crusting was advised to be performed two weeks later. A follow-up consultation with an ophthalmologist was scheduled for the day after surgery and was repeated as per the ophthalmologist’s recommendations. Additionally, an endocrinology consultation was arranged during and one week after surgery. Patients received L-thyroxin replacement therapy post-surgery to maintain normal serum levels of thyroid hormones after total thyroidectomy.

Case presentation

First Case

**Figure 1:**
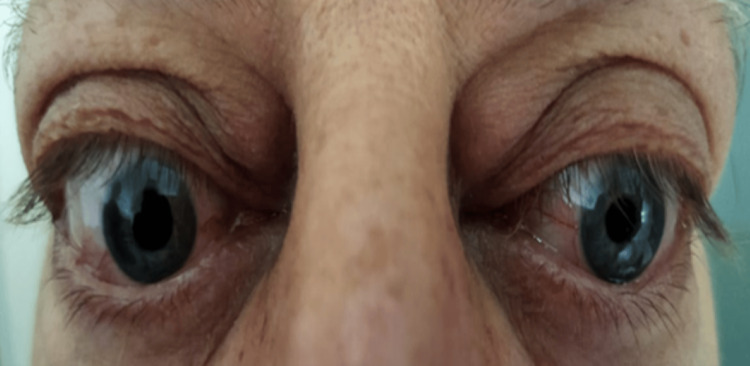
Bilateral proptosis with eyelid retraction, erythema and edema of the eyelids Photo courtesy to the ENT, head and neck surgery department of Hassan II University Hospital of Fez, Morocco, taken during consultation

**Figure 2:**
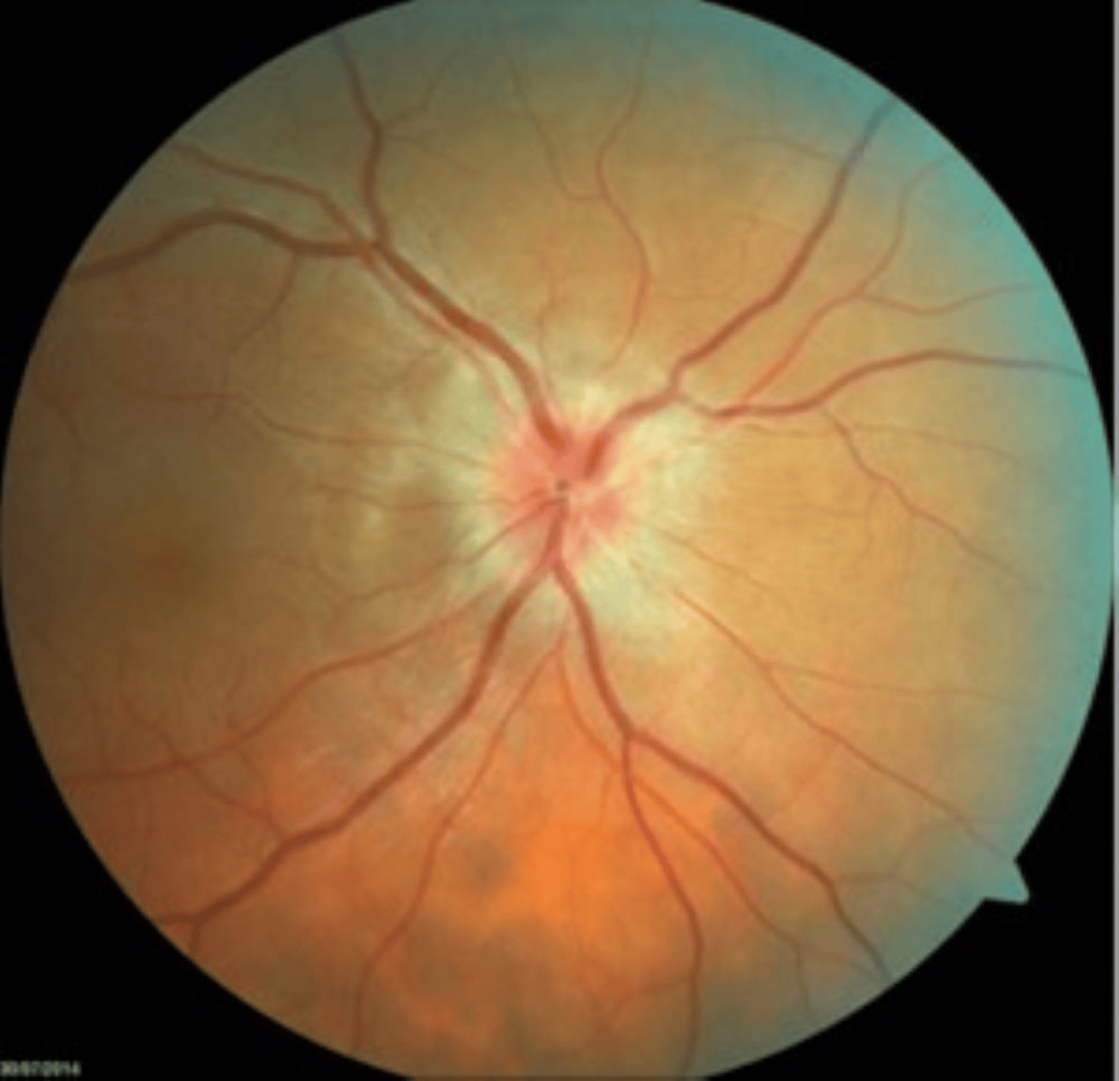
A pale retina in the fundoscopic exam Photo courtesy to the ENT, head and neck surgery department of Hassan II University Hospital of Fez, Morocco, taken during the preoperative period)

**Figure 3:**
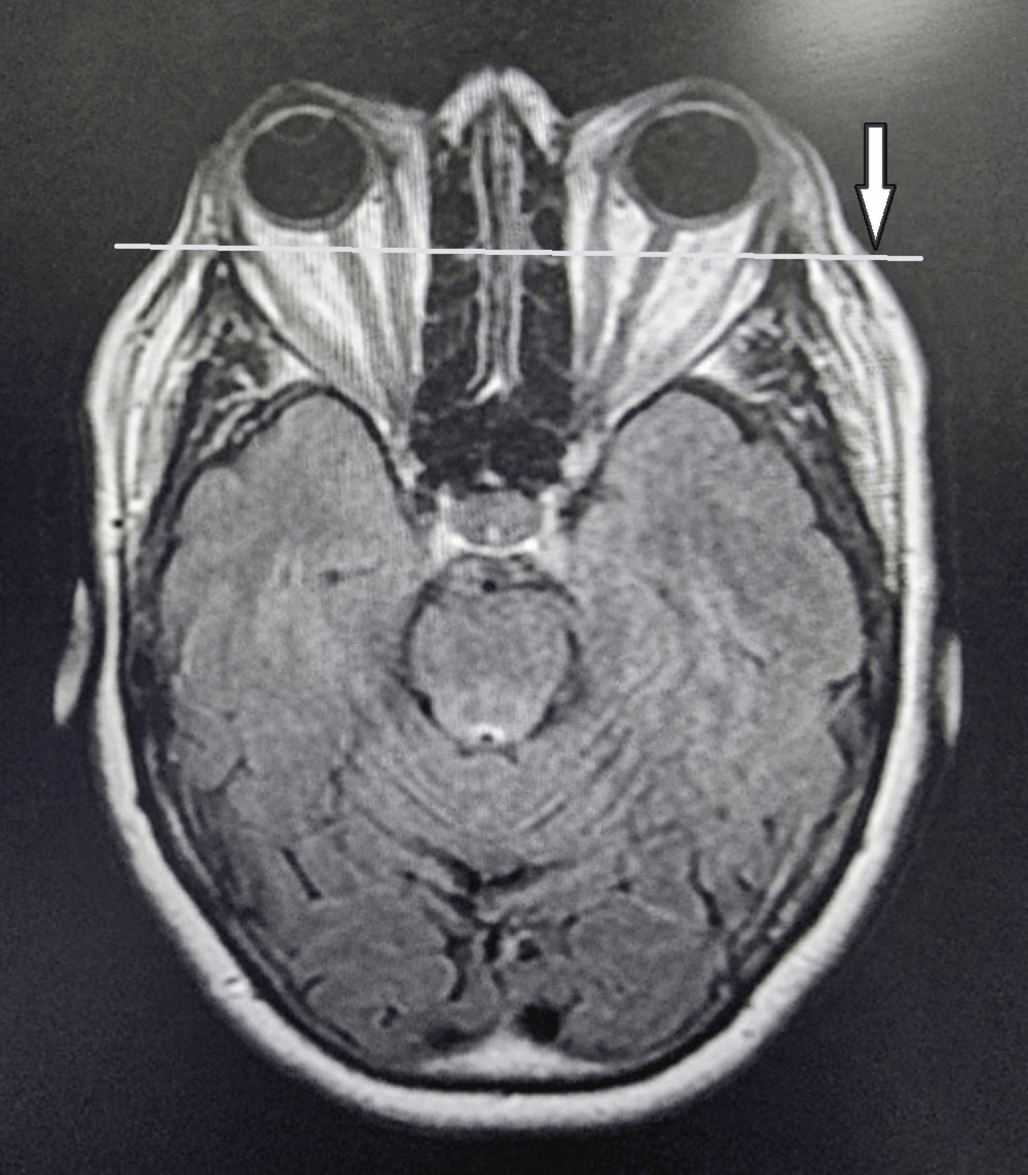
Orbital MRI axial view showing bilateral proptosis (white arrow) Image provided by the ENT, head and neck surgery department of Hassan II University Hospital of Fez, Morocco, taken during consultation

**Figure 4:**
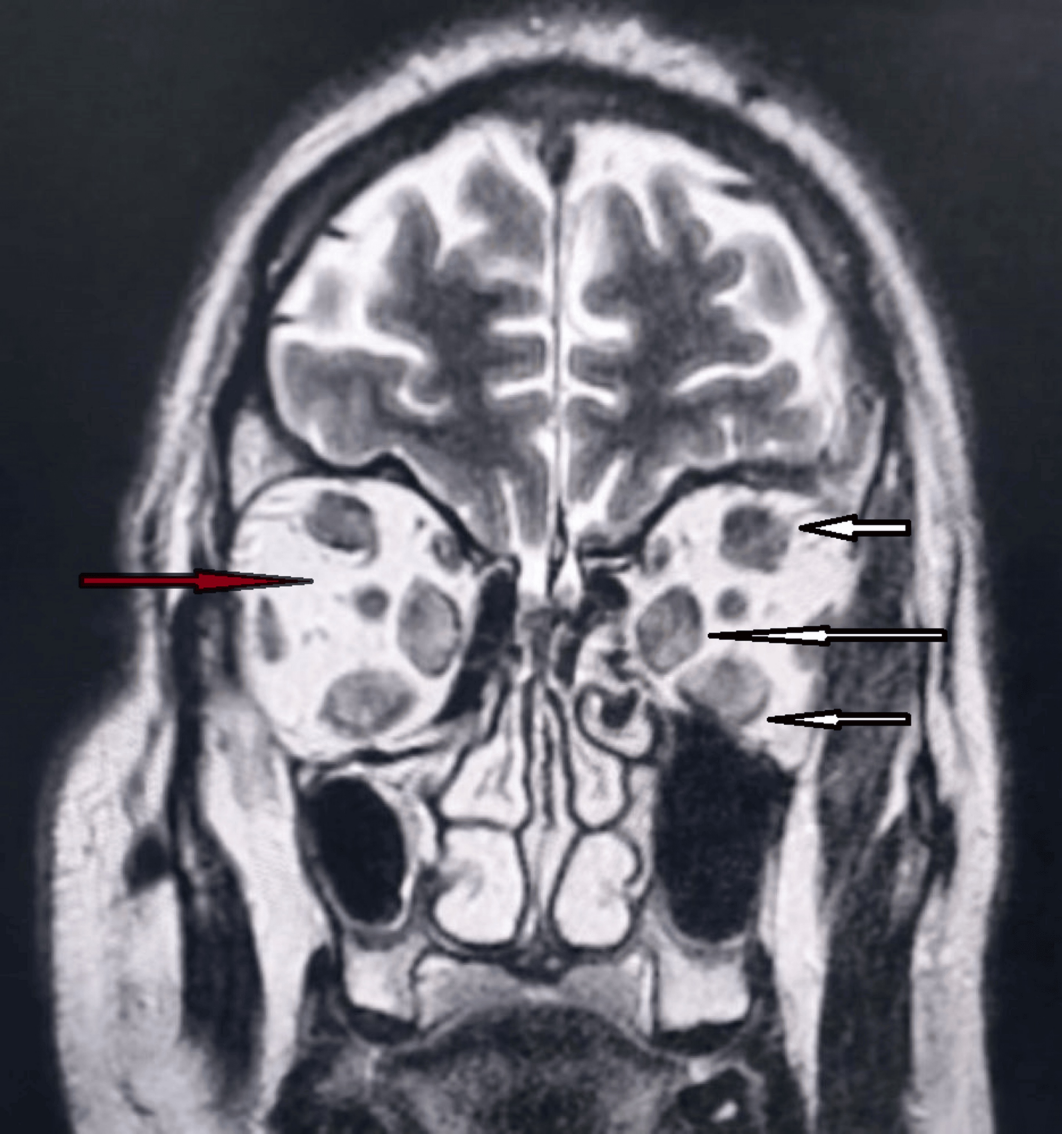
Orbital MRI coronal view showed an enlargement of the internal, inferior, and superior extraocular muscles in both eyes (white arrows) and orbital fat hypertrophy (brown arrow) Image provided by the ENT, head and neck surgery department of Hassan II University Hospital of Fez, Morocco, taken during consultation

**Figure 5:**
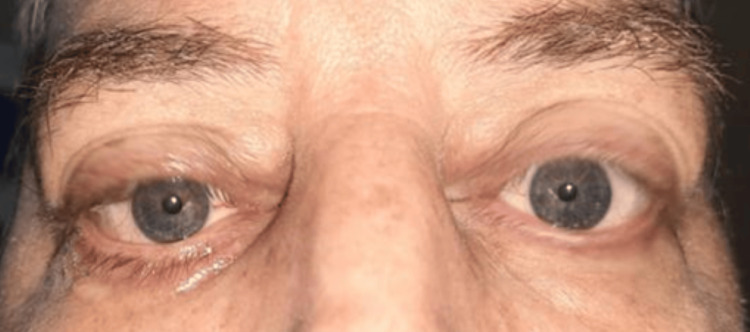
The clinical outcome after one month of the decompression surgery Photo courtesy of the ENT, head and neck surgery department of Hassan II University Hospital of Fez, Morocco, taken during the follow-up period

Our department received a referral for a 52-year-old male patient who had a 30-year smoking history and had been suffering from Graves' disease for 20 months. He complained of bilateral proptosis, significant visual acuity loss, and orbital pain, as well as moderate eyelid edema and retraction in both eyes. Eye examination showed conjunctival hyperemia, chemosis, and inflammation of the tear wattle in both eyes. Physical examination revealed marked bilateral proptosis, with the left eye being more affected (22 mm in the right eye, 26 mm in the left eye), eyelid retraction, erythema and edema of the eyelids, and mild orbital pain on palpation (Figure 1). The best corrected visual acuity was 5 out of 10 in both eyes, and the IOP was very high (38 mm Hg in the right eye and 40 mm Hg in the left eye). Fundus evaluation revealed a pale retina in both eyes (Figure 2). Contrast CT scans and MRI showed stage II proptosis in the right eye and stage III in the left eye (Figure 3), with enlargement of the internal, inferior, and superior extraocular muscles in both eyes and orbital fat hypertrophy (Figure 4). Moreover, the serum analysis revealed normal thyroid levels. The patient received IV corticosteroids, non-steroid anti-inflammatory medications, mydriatics, and lubricants and was advised to stop smoking, but the symptoms did not improve. The patient underwent an emergency endoscopic endonasal orbital and optic nerve decompression combined with an external approach for lateral wall decompression and total thyroidectomy, at the same time, which was successful with no thyroid surgery complications. Postoperative eye examination on the next day after surgery revealed visual acuity of 7/10 and ocular tonus of 17 OD/20 OG, which was further managed with topical medication. One month later, the IOP was normal, and the visual acuity was 8/10 in the right eye and 7/10 in the left eye (Figure 5).

Second Case

**Figure 6:**
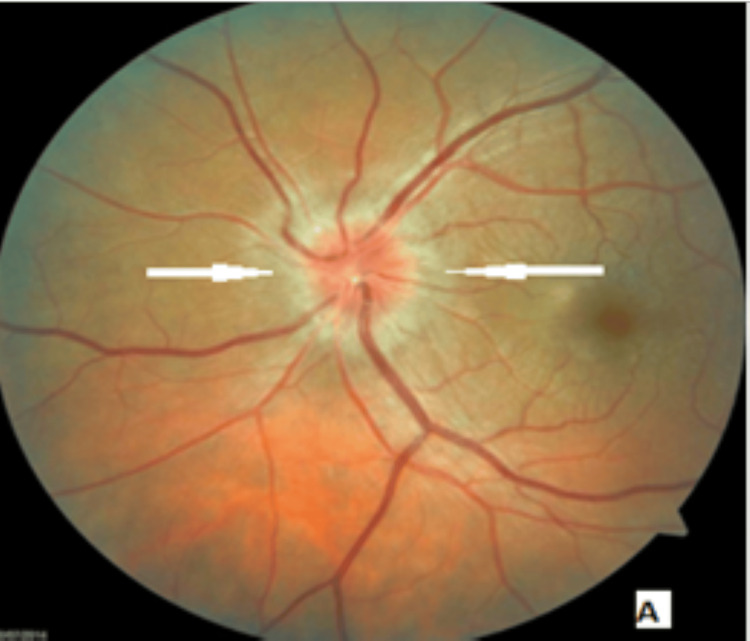
Fundus examination revealed a stage II papilledema Photo courtesy to the ENT, head and neck surgery department of Hassan II University Hospital of Fez, Morocco, taken during the preoperative period

A 73-year-old female patient with a two-year history of Graves’ disease and a family history of goiter and hyperthyroidism was referred to our department for bilateral proptosis, severe decrease in visual acuity, and moderate eyelid retraction and edema affecting both eyes. Eye examination showed conjunctive hyperemia, chemosis, and mild inflammation of the tears wattle in both eyes. Physical examination revealed bilateral marked proptosis mainly affecting the left eye (23 mm in the right eye, 28 mm in the left eye), eyelid retraction, erythema, and edema of the eyelids with limited eye motility. The best corrected visual acuity was 1 out of 10 in the left eye and 6 out of 10 in the right eye. The IOP was 25 mmHg in both eyes. Fundus evaluation revealed a stage II papilledema on both sides (Figure 6).

**Figure 7:**
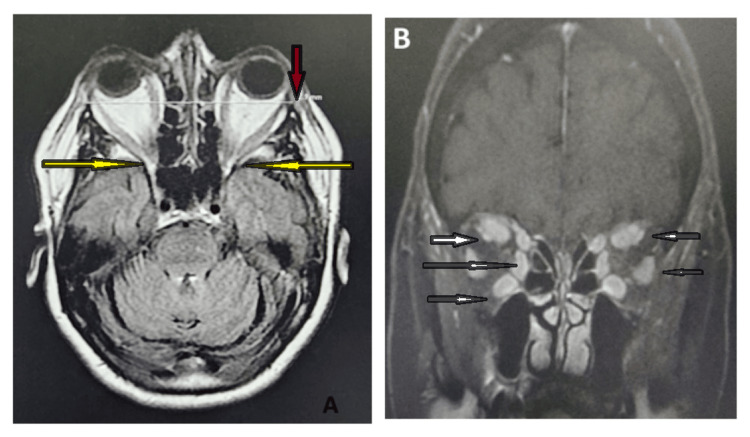
Orbital MRI axial and coronal view showed bilateral proptosis (brown arrow), an enlargement of all the extraocular muscles with an orbital fat hypertrophy (white arrows) and a stretching of the optic nerve on both sides (yellow arrows) Image provided by the ENT, head and neck surgery department of Hassan II University Hospital of Fez, Morocco, taken during consultation

Contrast CT scans and MRI showed a bilateral stage III proptosis with an enlargement of all the extraocular muscles on both sides, orbital fat hypertrophy, and stretching of the optic nerve on both sides (Figures 7A, 7B). Furthermore, the thyroid serum levels were within normal range. The patient received IV corticosteroids bolus, non-steroid anti-inflammatory medications, mydriatics, and lubricants with eyes patching during the night. However, symptoms did not improve after the initial treatment, and the visual acuity worsened. Subsequently, the patient underwent medial and inferior orbital walls’ endoscopic decompression surgery along with an urgent optic nerve decompression as well as a total thyroidectomy within the same day.

**Figure 8:**
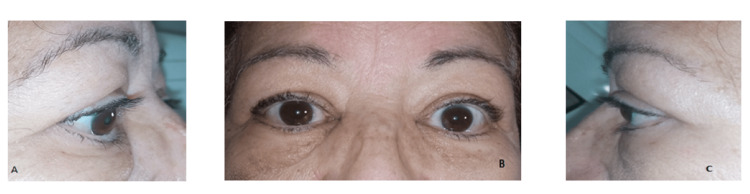
Regression of the proptosis on both sides after decompression surgery on the front and profile view Photo courtesy of the ENT, head and neck surgery department of Hassan II University Hospital of Fez, Morocco, taken during the follow-up period

In the few days after the surgery, the examination revealed a visual acuity of 2/10 compared to 1/10 before surgery in the left eye and 7/10 compared to 6/10 preoperatively in the right eye. However, after one month, it was estimated at 9/10 compared to the initial 6/10 in the right eye and at 5/10 compared to the initial 1/10 in the left eye with normal IOP on both sides (Figures 8A-8C).

## Discussion

Thyroid-associated orbitopathy (TAO) is an autoimmune condition that tends to affect patients primarily diagnosed with Graves’ disease (approximately 90%). However, it can also occur in patients with Hashimoto thyroiditis (about 3%) and primary hypothyroidism (around 1%) and in a small proportion without any apparent thyroid abnormalities (6%) [9]. The correlation between goiter and orbitopathy was initially described by Parry [9]. However, the connection between different orbital abnormalities and hyperthyroidism was identified by Graves himself, leading to the nomenclature “Graves’ orbitopathy.”

Risk factors contributing to the development of TAO include female gender, smoking, similar familial cases, stress, and ineffectively managed hypothyroidism following radioactive iodine therapy. Male gender, advanced age, and a rapid onset of orbitopathy are often associated with severe forms [2]. The presence of autoantibodies that target the thyrotropin receptor on thyroid follicular endothelial cells leads to excessive production of thyroid hormones, which primarily explains the hyperthyroidism observed in Graves’ disease [10]. Considering that all patients with GO eventually possess anti-thyrotropin-receptor antibodies, it is believed that the immune response attacking the thyrotropin receptor underlies both hyperthyroidism and TAO [11]. These antibodies also bind to CD40+ receptors expressed by fibroblasts in the orbit’s fat tissue and striated muscles, leading to inflammation and a significant increase in the production of hyaluronan and glycosaminoglycan. Consequently, fibroblasts transform into myocytes or adipocytes, resulting in an enlargement of the extraocular muscles or fat within the orbit. This leads to increased pressure within the rigid bony orbit and manifests as various forms of TAO, ranging from mild to severe or sight-threatening cases [2,10,11].

The clinical presentation of GO varies depending on the specific type of orbital tissues involved. When the inflammatory process predominantly affects the orbital fat tissue, patients, especially young females, typically experience a slow progression marked by eyelid retraction, proptosis, and ocular exposure [9]. Conversely, enlargement of the muscular orbital structures is linked to smoking and a family history of the disease, often impacting older individuals with higher severity. The expansion of the extraocular muscles results in restricted ocular movements, diplopia, edema, congestion of eyelids and conjunctiva, and potential optic nerve impairment due to thyroid dysfunction [9].

GO typically progresses through two distinct phases: an initial intense inflammatory period, followed by a persistent fibrotic stage, lasting approximately 6 to 18 months [12]. As the first phase subsides, a fibrotic stage begins. This stage is characterized by a static enlargement of the extra-ocular muscles and an excess of orbital fat.

During the acute phase, local measures such as eyelid tapes and lubrication are crucial to prevent corneal exposure, infection, or ulceration. Systemic corticosteroids may also be used to reduce inflammation and decrease complications. However, due to the multitude of side effects associated with long use of steroids and the high chances of recurrent episodes following treatment cessation, they are usually employed as a temporarily or in conjunction with surgical approaches. Low-dose radiotherapy may as well be considered during the acute stage in order to counteract the inflammation, although its effectiveness remains controversial [13]. Furthermore, quitting smoking is crucial given that cigarette smoking significantly increases the risk of GO, with an odds ratio among smokers vs. nonsmokers of 7.7. The risk is proportional to the number of cigarettes smoked every day [13].

Surgical intervention is rarely performed during the acute stage unless there is a specific threat to vision such as an exophthalmos with corneal exposure and keratitis, an increased orbital pressure, or compressive optic neuropathy non-responsive to medical treatments [14]. Fortunately, severe orbital disease is moderately uncommon, posing a risk to vision in only 3 to 5% of GO cases [6]. However, during the second phase, decompressive surgery is commonly considered if indications persist.

Various surgical techniques for optic nerve decompression are described in the literature. However, the endoscopic endonasal approach is gaining popularity due to its minimally invasive nature and excellent exposure of the optic canal and orbital apex [13]. Kennedy et al. [15] and Michel et al. [16] first portrayed the procedure in the early 1990s. The improved vision of key anatomic landmarks amid the endoscopic approach enables secure and intensive decompression of the whole inner orbital wall and medial portion of the orbit's floor, improving vision in the critical area of decompression in patients with optic neuropathy. This procedure has replaced the Walsh-Ogura procedure, which was the primary method of orbital decompression in the 20th century.

New-onset diplopia or worsening of pre-existing diplopia is the most common complication following orbital decompression surgery, occurring in 15 to 63% of patients [12]. Patients should be warned about the possibility of diplopia persisting or worsening after decompression, necessitating potential strabismus surgery. The incidence of postoperative epistaxis is comparable to that of routine endoscopic sinus surgery. It is usually caused by bleeding from the posterior remnant of the middle turbinate and can be effectively managed through direct cauterization of the bleeding site using endoscopic control [12]. Orbital hematoma is rare because the evacuation of the periorbital bone and fascia avoids localized blood accumulation. Postoperative infections are reduced through preoperative measures and postoperative antibiotics. Epiphora may develop if the maxillary antrostomy is extended too far anteriorly, resulting in transection of the nasolacrimal duct. However, this can be effectively managed with an endoscopic dacryocystorhinostomy. Leakage of cerebrospinal fluid (CSF) and visual deficits are exceptionally uncommon complications, mostly reported after non-endoscopic decompressive methods [13].

Optic nerve decompression procedures are standardized globally. However, certain measures should be considered to ensure optimal outcomes. Before surgery, a visual assessment is performed, which includes bilateral visual acuity, fundoscopy, and computerized visual field testing. Additionally, craniofacial CT and/or MRI are used to meticulously study the area's anatomy and select appropriate candidates. They are also used to anticipate any surgical difficulties, such as nasal turbinate hypertrophy, septal deviation, sphenoid sinus pneumatization and septa, Optic Canal length, hyperostosis, and internal carotid artery (ICA) position. Thorough preoperative preparation is essential to prevent excessive bleeding and infection [17].

During the surgery, it is essential to carefully open the Onodi cell to prevent any Optic Nerve injury, identify the ethmoidal arteries with caution, and keep the bone of the C5 paraclinoid portion of the ICA canal intact to reduce the vascular risk when opening the inferior part of the optic canal. In some cases, carotid arteries and/or optic nerves are not covered by bone, and Doppler control can be useful in locating these important vessels. The dura mater of the anterior skull base is fragile in various locations and must be pushed away, especially when drilling, to avoid any CSF leak [17].

Finally, the patient should be aware of potential visual, infectious, and vascular risks and rhinological discomfort. They should also be educated about what to avoid during the first few days, such as coughing, sneezing, or blowing their nose, as these activities may lead to exophthalmia caused by air getting into the orbit.

Despite the technical advances in GO management, its impact on patients’ quality of life remains notable, even in milder forms [18,19]. Moreover, it presents a significant public health challenge due to both direct and indirect costs incurred [19].

## Conclusions

Treating GO, especially in its severe forms, can be a major therapeutic challenge, as currently available treatments are often ineffective. Therefore, a multidisciplinary approach is needed to manage the disease and its complications. Endoscopic endonasal optic nerve decompression, is a safe, effective, and minimally invasive technique for treating compressive optic nerve forms of GO that threaten sight. It offers an encouraging functional result.
